# Therapeutic lumbar puncture for headache in idiopathic intracranial hypertension: Minimal gain, is it worth the pain?

**DOI:** 10.1177/0333102418782192

**Published:** 2018-06-17

**Authors:** Andreas Yiangou, James Mitchell, Keira Annie Markey, William Scotton, Peter Nightingale, Hannah Botfield, Ryan Ottridge, Susan P Mollan, Alexandra J Sinclair

**Affiliations:** 1 Metabolic Neurology, Institute of Metabolism and Systems Research, College of Medical and Dental Sciences, University of Birmingham, Birmingham, UK; 2 Centre for Endocrinology, Diabetes and Metabolism, Birmingham Health Partners, Birmingham, UK; 3 Department of Neurology, University Hospitals Birmingham NHS Foundation Trust, Birmingham, UK; 4 NIHR/Wellcome Trust Clinical Research Facility, University Hospitals Birmingham NHS Foundation Trust, Birmingham, UK; 5 Birmingham Clinical Trials Unit, College of Medical and Dental Sciences, University of Birmingham, Birmingham, UK; 6 Birmingham Neuro-Ophthalmology Unit, Ophthalmology Department, University Hospitals Birmingham NHS Foundation Trust, Birmingham, UK

**Keywords:** Idiopathic intracranial hypertension, headache, lumbar puncture, post lumbar puncture headache

## Abstract

**Background:**

Headache is disabling and prevalent in idiopathic intracranial hypertension. Therapeutic lumbar punctures may be considered to manage headache. This study evaluated the acute effect of lumbar punctures on headache severity. Additionally, the effect of lumbar puncture pressure on post-lumbar puncture headache was evaluated.

**Methods:**

Active idiopathic intracranial hypertension patients were prospectively recruited to a cohort study, lumbar puncture pressure and papilloedema grade were noted. Headache severity was recorded using a numeric rating scale (NRS) 0–10, pre-lumbar puncture and following lumbar puncture at 1, 4 and 6 hours and daily for 7 days.

**Results:**

Fifty two patients were recruited (mean lumbar puncture opening pressure 32 (28–37 cmCSF). At any point in the week post-lumbar puncture, headache severity improved in 71% (but a small reduction of −1.1 ± 2.6 numeric rating scale) and exacerbated in 64%, with 30% experiencing a severe exacerbation ≥ 4 numeric rating scale. Therapeutic lumbar punctures are typically considered in idiopathic intracranial hypertension patients with severe headaches (numeric rating scale ≥ 7). In this cohort, the likelihood of improvement was 92% (a modest reduction of headache pain by −3.0 ± 2.8 numeric rating scale,* p* = 0.012, day 7), while 33% deteriorated. Idiopathic intracranial hypertension patients with mild (numeric rating scale 1–3) or no headache (on the day of lumbar puncture, prior to lumbar puncture) had a high risk of post- lumbar puncture headache exacerbation (81% and 67% respectively). Importantly, there was no relationship between lumbar puncture opening pressure and headache response after lumbar puncture.

**Conclusion:**

Following lumbar puncture, the majority of idiopathic intracranial hypertension patients experience some improvement, but the benefit is small and post-lumbar puncture headache exacerbation is common, and in some prolonged and severe. Lumbar puncture pressure does not influence the post-lumbar puncture headache.

## Background

Idiopathic intracranial hypertension (IIH) is a disease of unknown aetiology typically manifesting in obese females aged 20 to 40 (1,2). There is a risk of severe visual loss occurring (in up to one quarter of patients (3)) but for the majority of patients the day-to-day reality is one of a chronic disease, dominated by disabling headaches which significantly reduce quality of life (4–6). The incidence of IIH appears to be rising with global obesity rates and many patients will have repeated hospital admissions for assessment and treatment (7–10).

Lumbar punctures (LPs) occur at diagnosis and are sometimes repeated during the course of the illness to evaluate disease status. Some centres also perform therapeutic LPs to manage headache pain (11). Complications of LPs are well documented and include local discomfort, post-dural puncture headache, severe anxiety and discomfort, and more rarely cerebrospinal fluid (CSF) infection, localised haematoma and paraparesis (12,13). There is also increasing awareness of the emotional costs and procedure-induced anxiety noted by IIH patients. We have also observed post-LP headache exacerbation in IIH patients, which can precipitate a further medical consultation, attendance at the hospital emergency department, and even hospital admission. The risk of headache exacerbation post-LP has not been previously characterised in IIH.

There is a lack of prospective data describing the improvement and exacerbations in IIH headaches in the week post-LP. This study aimed to evaluate the temporal change in headache severity in the week following a standardised LP, in patients with active IIH. Furthermore, we aimed to stratify the response to LP by baseline headache severity and evaluate the extent of improvement and the likelihood of an exacerbation. Importantly, we also sought to define the influence of LP opening pressure on post-LP headache.

## Methods

### Participants

Patients with IIH were prospectively recruited from the out-patients department at University Hospitals Birmingham NHS Foundation Trust, a United Kingdom tertiary referral hospital. Patients were asked to consent to LP for research purposes. Eligibility criteria included fulfilment of the updated modified Dandy criteria (no evidence of intracranial pathology or venous sinus thrombosis (magnetic resonance or computerised tomography imaging and venography at diagnosis) and LP opening pressure ≥ 25 cmCSF) (14). Consequently, all patients had undergone a previous diagnostic LP. Only those with active IIH (papilloedema with a Frisén grade ≥ 1 and LP opening pressure ≥ 25 cmCSF) at the time of the study recruitment were included (14,15). To avoid recruitment bias, all those prospectively screened that met the eligibility criteria were informed of the study and offered recruitment.

### Patient consents

Patients gave written informed consent to participate in the study. The study was conducted according to the Declaration of Helsinki and approval was obtained through Research Ethics Committees (13/YH/0366 and 14/WM/0011).

### Patient assessment

Eligibility was confirmed following assessment by a neuro-ophthalmological examination, which included fundus photography with subsequent grading of the papilloedema (Frisén grade 0–5, most severe) performed by three masked reviewers. Patient demographics were recorded.

A standardised LP was performed: A Quincke type point, 20GA 3.50 IN, 0.9 mm × 90 mm (Becton Dickinson™, Spain) spinal needle was used. An ultrasound scan was used to mark the LP position with measurement of the CSF depth from the skin recorded. LPs were performed in the left lateral decubitus position and, following dural puncture, legs were repositioned to mitigate against compressing the abdomen. A stabilised reading was recorded once any fluctuations in the manometer settled. After measurement of the opening pressure, a standardised volume of CSF was drained (estimated at 10 ml) and finally a closing pressure was recorded. CSF red cell count was evaluated in the University Hospital Birmingham NHS Foundation Trust laboratory.

### Headache evaluation

Headache phenotype was characterised according to the International Headache Society ICHD-3 beta classification at baseline by a headache specialist (16). Headache preventative medications, acetazolamide use and acute analgesic use on the day of LP were recorded. Headache severity was evaluated using a Numeric Rating Scale (NRS, 0 (no pain) to 10 (most severe pain)). Headache severity NRS was prospectively recorded just prior to LP at 0 hours and following the LP at 1, 4 and 6 hours and daily for 7 days post-LP using a paper diary. The paper headache diary was returned by post 1 week after the LP, this was facilitated by a telephone reminder from the research nurse. The headache severity NRS was further categorised into mild (1–3) moderate (4–6) severe (7–10) pain severity (17). We defined those with a change in the headache severity score by ≥ 4 as a severe exacerbation.

### Statistical analysis

Statistical analyses were undertaken on SPSS, Armonk, NY: IBM Corp. Version 24.0 (2016). The characteristics were generally described as median and interquartile range (IQR) (non-parametric data) and headache severity NRS as mean with standard deviation unless otherwise stated. Mann–Whitney U test quantified changes of clinical characteristics of patients that had follow-up visits and the ones that deteriorated. Wilcoxon signed-rank test and Friedman test were used to compare changes in headache severity at different time points. Spearman rank correlation quantified the association between categorical variables and headache severity. Data were further evaluated using a multiple linear regression model. A Bonferroni correction was applied to account for multiple analyses. Only patients with headache diary data that included baseline, 1 hour and 7 days were included for analysis. Where data was missing at other time points or for other variables studied, this was noted in the results. Statistical significance was considered at *p* < 0.05 level (two-tailed) unless otherwise stated.

## Results

Seventy headache diaries were returned. Four diaries had insufficient data for analysis and were excluded. The analysed cohort comprised 52 patients, and of these 14 had second lumbar punctures performed (Supplementary Table 1). Headache phenotype was characterised at baseline; in this study, all patients had active IIH, thus headaches were not primary headaches. However, based on phenotypic characteristics using the International Headache Society ICHD-3 beta classification, we noted: Migraine-like or probable migraine-like (n = 53, 80%), headache attributed to IIH (n = 23, 35%), tension-type headache-like (n = 5, 8%), other (n = 5, 8%) and not classifiable (n = 7, 11%) (Table 1). Some participants had more than one headache phenotype. We found no significant difference in the baseline characteristics or headache outcomes following the first and repeat LP. The mean headache score pre-LP was 3.6 ± 2.8 on the NRS; 18% (12/66) had severe headaches, 35% (23/66) had moderate headaches, 24% (16/66) had mild headaches and 23% (15/66) had no headache on the day of the LP, at the pre-LP time point.

**Table 1.** Baseline characteristics for the whole cohort including data from all patient visits (n = 66). Data presented as median, interquartile range (IQR) or percentage (visits) where specified.

Characteristic	Median (interquartile range)
Age at baseline	31 (25–35)
BMI (kg/m^2^)	39 (35–47)
Weight (kg)	107 (93–120)
Pre-LP headache severity (NRS)	4 (1–6) (mean 3.6 (SD: 2.8))
LP opening (cmCSF)	32 (28–37)
LP closing (cmCSF)	19 (17–21)
Amount CSF drained (ml)	10 (10–12)
RBC in CSF (cmm)^#^	7 (1–36)
Depth from skin to CSF (mm) as measured by ultrasound^##^	67 (61–74)
Frisén Grade	Right eye 2 (1–2) Left eye 2 (1–3)
	Percentage (visits)
Ethnicity	
White	91% (60)
Mixed/multiple ethnic group	5% (3)
Asian/Asian British	3% (2)
Black/African/Caribbean	2% (1)
Analgesic on day^###^	24% (11)
Headache preventative therapy	24% (16)
Acetazolamide	27% (18)
Headache phenotypes*	
Migraine-like and probable migraine-like	80% (53)
Attributed to IIH	35% (23)
Tension type-like	14% (9)
Other	8% (5)
Not classifiable	11% (7)

Note: Where data is missing, the number of visits included is indicated as ^#^ = 61, ^##^ = 22, ^###^ = 46.

* Patients may have experienced more than one headache phenotype at baseline. Headaches exist in the setting of active IIH thus are not a primary headache; the phenotype of the headaches are classified according to the ICHD-3 beta.

Acetazolamide therapy was documented in 27% of the patients with a median dose of 500 mg and range 250–1500 mg. No other diuretics were used. In all, 24% used acute analgesics, of which 73% had paracetamol, 18% ibuprofen and 9% tramadol. One or more headache preventatives were used by 24% of the patients: 63% topiramate, 44% amitriptyline, 13% gabapentin, 6% mirtazepine and 6% propranolol.

### Therapeutic response to lumbar puncture

**Figure 1.**
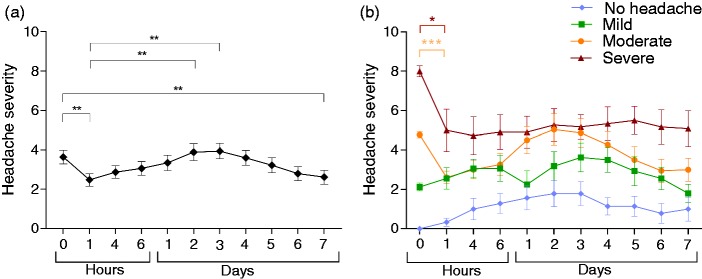
Headache severity following lumbar puncture, mean ± standard error of the mean (SEM). (a) Whole cohort; (b) cohort classified by baseline headache severity. NRS: numeric rating scale. *p < 0.05, **p < 0.01, ***p < 0.001 (Wilcoxon signed-rank test).

Seventy one percent improved at some point in the week post-LP, with the greatest reduction in headache severity occurring at 1 hour post-LP, but this was a small improvement (−1.1 ± 2.6 NRS, *p* < 0.001), this was maintained at 7 days (−1.0 ± 2.7 NRS, *p* = 0.004) (Table 2, Figure 1(a),(b), Supplementary Figure 1(a)). A more dramatic improvement was less common, with an improvement by greater than 4 on the NRS noted in 32%. Twenty three percent of these patients experienced an improvement that lasted at least two consecutive days (Table 2).

**Table 2.** Pre-LP headache category and severity of the headache on the numeric rating scale (NRS) as categorised into mild (1–3) moderate (4–6) severe (7–10) pain. It illustrates the % (number) of patients that had improvement in their headache at any point in the week post-lumbar puncture (LP).

Pre-LP category	% (number)	Improvement by ≥4 on the NRS, % (number)	Improvement by ≥4 for ≥2 days on the NRS, % (number)
All (n = 66)	71 (47)	32 (21)	23 (15)
All with headache (n = 51)	92 (47)	41 (21)	29 (15)
Those with no headache (n = 15)*	N/A	N/A	N/A
Mild (n = 16)	81 (13)	0	0
Moderate (n = 23)	100 (23)	61 (14)	43 (10)
Severe (n = 12)	92 (11)	58 (7)	42 (5)

NRS: Numeric Rating Scale; N/A: Not applicable.

* On the day of the LP, at the pre-LP timepoint.

A sensitivity analysis, excluding these, revealed that the number of patients experiencing an improvement was 92%, but the extent of the improvement remained small (−1.5 ± 2.8 NRS, *p* < 0.001 at 1 hour and by 7 days −1.5 ± 2.5 NRS, *p* < 0.001) (Table 3, Supplementary Figure 1(b), 1(c)).

**Table 3.** Number of patients experiencing an improvement or deterioration in headache at 1 hour and 7 days post-lumbar puncture (LP) compared to baseline. The *p*-values indicate the change between pre-LP and 1 hour or 7 days post-LP.

Baseline	Improvement % of patients (number)	Deterioration % of patients (number)	Change in headache score (NRS) mean ± SD	*p*-value**
*Changes at 1 hour post-LP*				
All (n = 66)	58 (38)	20 (13)	−1.1 ± 2.6	0.001
All with headache (n = 51)	75 (38)	20 (10)	−1.5 ± 2.8	<0.001
No headache (n = 15)*	0	20 (3)	0.3 ± 0.8	0.102
Mild (n = 16)	50 (8)	50 (8)	0.4 ± 2.5	0.713
Moderate (n = 23)	91 (21)	4 (1)	−2.2 ± 1.6	<0.001
Severe (n = 12)	75 (9)	8 (1)	−3.0 ± 3.7	0.024
*Changes at 7 days post-LP*				
All (n = 66)	47 (31)	18 (12)	−1.0 ± 2.7	0.004
All with headache (n = 51)	61 (31)	16 (8)	−1.5 ± 2.5	<0.001
No Headache (n = 15)	0	27 (4)	1.0 ± 2.3	0.066
Mild (n = 16)	56 (9)	19 (3)	−0.3 ± 2	0.316
Moderate (n = 23)	61 (14)	22 (5)	−1.7 ± 2.3	0.007
Severe (n = 12)	67 (8)	0 (0)	−3.0 ± 2.8	0.012

NRS: Numeric rating scale.

* On the day of the LP, at the pre-LP timepoint, **Wilcoxon signed-rank test.

### Headache exacerbation post lumbar puncture

Sixty four percent experienced a headache exacerbation at some point in the week following LP (Table 4). Of note, a severe exacerbation by ≥ 4 points on the NRS occurred in 30%. In 20%, this severe deterioration lasted 2 days or more. Rates of improvement or deterioration in the week after LP vary with baseline headache severity (Tables 2, 4, Supplementary Figure 1). Deterioration was most evident at day 2 and 3 (compared to the 1-hour time point, mean increase at day 2 of 1.5 ± 3.4 NRS, *p* = 0.002 and at day 3, mean increase of 1.5 ± 3.1 NRS, *p* = 0.001) (Figure 1(a)).

**Table 4.** Pre-LP headache category and severity of the headache on the numeric rating scale (NRS) as categorised into mild (1–3) moderate (4–6) severe (7–10) pain. This illustrates the % (number) of patients that had deterioration in their headache at any point in the week post-lumbar puncture (LP).

Pre-LP category	% (number)	Deterioration by ≥4 on the NRS, % (number)	Deterioration by ≥4 for ≥2 days on the NRS, % (number)
All (n = 66)	64 (42)	30 (20)	20 (13)
All with headache (n = 51)	63 (32)	27 (14)	16 (8)
Those with no headache (n = 15)*	67 (10)	40 (6)	31 (5)
Mild (n = 16)	81 (13)	50 (8)	31 (5)
Moderate (n = 23)	65 (15)	26 (6)	13 (3)
Severe (n = 12)	33 (4)	N/A	N/A

N/A: not applicable.

* On the day of the LP, at the pre-LP timepoint.

### Lumbar puncture outcomes analysed by baseline headache severity

#### Severe headache pre-LP

**Figure 2.**
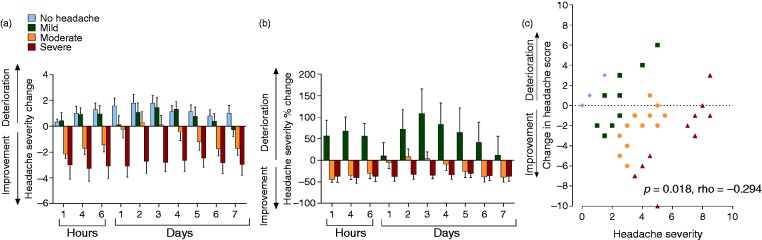
Changes in headache severity score (defined by the numerical rating scale (NRS)) post-LP. (a) Change in headache severity score, data as mean ± SEM; (b) percentage change in headache severity, data as mean ± SEM; (c) correlation of change in headache severity between baseline and 1 hour (y axis) with the mean pre-LP and 1 hour post-LP scores (x axis) using Oldham’s method, *p* = 0.018, rho = −0.294 (Spearman rank correlation).

Ninety two percent improved at some point in the week following LP, with 42% experiencing an improvement of ≥ 4 on the NRS lasting at least 2 days (Table 2). Deterioration was noted in 33% over the week (Table 4). The extent of improvement was −3.0 ± 3.7 NRS, *p* = 0.024 at 1 hour and −3.0 ± 2.8 NRS, *p* = 0.012 at 7 days (Table 3, Figures 1(b) and 2(a) and (b)).

#### Moderate headache pre-LP

In those with moderate headache pre-LP, improvement was noted in all (100%) patients with 61% experiencing an improvement ≥ 4 on the NRS lasting at least 2 days (Table 2). Deterioration was noted in 65% over the week and 26% deteriorated by ≥ 4 points on the NRS (Table 4).

The patients most likely to have improved at 1 hour were those with moderate headache pre-LP (91% with mean reduction of −2.2 ± 1.6 NRS, *p* < 0.001) (Table 3, Figures 1(b), 2(a), 2(b)). At 7 days, 61% of the patients improved with mean improvement −1.7 ± 2.3 NRS (Table 3, Figures 1(b), 2(a), 2(b)).

#### Mild headache pre-LP

In those with mild headache pre-LP, improvement was noted in 81% (Table 2). Deterioration following LP was most pronounced in those with mild headache pre-LP, with deterioration noted in 81% (Table 4). The chance of severe deterioration by ≥ 4 points on the NRS was 50% with 31% experiencing this for 2 days or more (Table 4). In this group we observed that 44% (7/16) experienced an exacerbation greater than seven on the NRS. In the majority, the exacerbation was observed between days 2–4 post-LP (Figures 1(b), Supplementary Figure 1(d)).

#### No headache pre-LP

There was no headache present on the day of the LP, at the pre-LP time point in 15 patients relating to the episodic nature of headaches in some IIH patients (6). For the patients that did not have a headache pre-LP, deterioration was noted in 67%. The chance of severe deterioration by ≥ 4 points on the NRS was 40%, with 31% experiencing this in 2 days or more (Table 4).

### Impact of lumbar puncture pressure and other disease and procedural variables

The LP opening pressure did not affect the post-LP headache severity. Furthermore, there was no relationship between the headache response post-LP and the BMI, height, depth from skin to CSF, Frisén papilloedema grade, LP closing pressure, number of LP attempts, CSF red blood cell count, acute analgesics use or the use of headache preventatives or acetazolamide. Further evaluation of those demonstrating a deterioration of ≥ 4 points on the NRS found no relationship with the above factors. Only the headache severity prior to LP showed an association with the headache severity (*p* < 0.018, rho = −0.294 at 1 hour and at 7 days (*p* < 0.001, rho = −0.402, (Figure 2(c), Oldham’s methods) (18). There was no relationship between headache phenotype and headache outcomes; however, this study was not powered for this subgroup analysis so meaningful conclusions cannot be drawn.

## Discussion

LPs maybe considered to relieve headache in patients with IIH; however, the efficacy and potential risk of post-LP headache exacerbation have not been previously defined. Whilst many patients and clinicians expect beneficial effects of LP on headache severity in IIH, this study has highlighted that this is not always the case.

We have demonstrated that although the majority of patients will experience an improvement in headache severity, the extent of the improvement is small, and the risk of a post-LP headache exacerbation is significant.

Overall, we noted that 71% of patients noted headache improvement after LP. This is comparable with the only other prospectively collected data in this area collected by the Danish Headache Center which noted improvement in 72% of subjects at 10–15 minutes post-LP (19). Of interest, our data evaluating headache severity over the week following LP noted that improvement was most marked by 1 hour post-LP and predicted the improvement at 7 days.

Improvement in headache post-LP was small (−1.1 ± 2.6 NRS at 1 hour and −1.0 ± 2.7 at 7 days). There is no consensus in the literature defining the minimally clinically important difference for a change in headache severity. In pain literature, with a change of greater than 2 in the score, NRS is judged to be clinically meaningful (20,21). Improvement post-LP was greatest in those with severe headache pre-LP (−3.0 ± 3.7 at 1 hour and −3.0 ± 2.8 at 7 days) and is likely to be more meaningful to patients. This is the subgroup with the greatest benefit from LP (92% improved). However, unfortunately this group were also susceptible to an exacerbation of their headache (33%) at some point in the week after LP.

Those patients with moderate headache (NRS 4–6) pre-LP also benefited (100% experienced an improvement but again the extent was small (−1.7 ± 2.3 NRS by 7 days)). However, the risk of a post-LP headache exacerbation was considerable (65%).

Headache severity prior to LP was the only factor that influenced the headache response post-LP. Consequently, those with less headache at the time of LP had the greatest chance of experiencing a post-LP headache exacerbation (81% in those with mild headache, NRS 1–3). Clearly, therapeutic LPs aiming to relieve headaches are much less likely to be considered in this group. However, knowledge of the risk of headache exacerbation post-LP is important when consenting patients undergo LP for diagnosis or assessment of disease activity. Post-LP headache exacerbation can lead to patients re-presenting to medical services following the LP. Of note, we have shown that 20% of IIH patients will experience a deterioration by ≥4 NRS that lasts at least two consecutive days.

The risk of headache exacerbation post-LP appears higher than that noted in non-IIH patients (64% of IIH versus 26% of non-IIH also with a traumatic needle) (22). This may be due to the limited period of monitoring post-LP in other studies. However, it is also possible that patients with intracranial pressure (ICP) dysregulation could be more susceptible to fluctuations in ICP.

The headache exacerbation post-LP described in this study cannot be reliably stated as low-pressure headache, as prolonged ICP monitoring was not utilised. We did collect diary data on postural aspects of the headache but did not feel this was reliable enough to draw firm conclusions. In clinical terms, it is the extent and rapidity of the headache alleviation on lying that can identify low pressure headaches after LP. The International Headache Classification (ICHD-3 beta) definition of post-dural puncture headache stipulates a headache that has developed within 5 days of dural puncture, which is not better accounted for by another ICHD-3 beta diagnosis (16). This definition does not specify a postural component or take into account the pre-LP headache. Consequently, the headache exacerbations noted in our study are all, by the ICHD-3 beta criteria, a post-dural puncture headache. However, we cannot be certain when differentiating a post-dural puncture headache from a recurrence of baseline headache phenotypes. Future studies could be more accurate in this differentiation by using a physician or specialist nurse to assess the headache phenotype at each time point or using an ICP monitor.

We had hypothesised that IIH disease activity (papilloedema grade and CSF opening pressure), patient parameters (weight, BMI, depth of CSF from skin as measured by ultrasound, use of acute analgesics, headache preventative or acetazolamide) and/or procedural factors (red cell count, number of LP attempts, CSF closing pressure) could influence headache outcomes after LP. In the settings of this study design this was not the case, with no relationship observed between any of these factors. Of particular interest was the lack of relationship between LP pressure and headache outcomes. In keeping with this, headache disability in IIH has been shown to be independent of LP pressure (6) and the observation that LP opening pressure is not related to headache response post-LP is of particular interest and is corroborated by others (19). This could suggest that the degree of ICP elevation is not the salient factor driving headache pain (19). Acetazolamide dosage in this study was mean 500 mg (range 250–1500 mg) and only used by 27%, consequently we cannot exclude whether higher acetazolamide doses could have impacted on headaches outcomes.

The mechanism underlying the fluctuation in IIH headache, post-LP, are not fully understood but may include that the LP could create a CSF leakage with ICP falling to a “normal” range for that patient, with consequent improvement, or ICP could fall below the “normal” range for that patient, with consequent deterioration in headache. Additionally, it is possible that alternations in ICP could influence the generation of migraine-like attacks.

Although the use of a standardised protocol for the LP was essential for the aims of this study, it did not allow us to investigate the effects of different needle types or the impact of draining different CSF volumes on headache severity. We acknowledge that the headache responses could have been influenced by patients’ previous experience of LPs and by the information relayed when consenting for LP (patients were aware that headaches could exacerbate or improve after LP in IIH patients). Additionally, conclusions cannot be drawn regarding the effect of LP on ophthalmic symptoms or signs, as these were not assessed. It would be of future interest to evaluate the role of LP in modifying visual outcomes in cases with fulminant IIH and rapid visual loss. Future studies evaluating patients over a longer period post-LP would be of interest. It would also be valuable to include a pre-LP headache diary to further characterise the pre-LP headaches more accurately. Additionally, evaluation of the effects of a sham LP would be of interest to enable further interpretation of the results. We acknowledge that the analysis of the cohort by headache subgroup reduced the power of the analysis but as the results were highly significant the impact of this analysis is unlikely to have changed the meaning of the results.

In this study, we have highlighted that the only factor which influences headache post-LP is pre-LP headache severity. LP pressure does not influence the post-LP headache. We have shown that after LP the majority of IIH patients experience some improvement over the following week (71%). But, of note, this benefit is small (mean reduction of one point on the NRS) and post-LP headache exacerbation is common (64% deteriorate at some point in the week post-LP), and in some cases this is prolonged and severe. However, the greater the headache severity pre-LP, the greater the likelihood of a therapeutic response to LP and the least chance of post-LP headache exacerbation.

We would recommend that therapeutic LPs to treat headache are only considered in those with severe (NRS 7–10) headaches at baseline, and patients should be aware that the improvements are modest over the following week (92% will improve but the mean improvement is a reduction of 3 on the NRS). If patients are undergoing LP during the course of their disease, the baseline headache severity should be noted when consenting the patient on the likelihood of a post-LP headache exacerbation: A third of those with no or mild headaches (NRS < 3) pre-LP will have a significant headache exacerbation (>4 on the NRS) lasting >2 days. This is of relevance, as headaches in IIH are frequently episodic (mean 12 days per month in the IIHTT (6)), and it is consequently possible that patients with disabling headaches may undergo LP on a day when they do not have a headache attack. This study does not allow us to comment on the utility of LP to modify or protect vision.

## Clinical implications

- Seventy one percent of IIH patients experience an improvement in headache at some point in the week post-LP.
- The extent of headache improvement is small (reduction by one point on the NRS), but greater in those with severe headaches, NRS ≥ 7, (reduction by three points on the NRS).
- Post-LP headache exacerbation is common in IIH (64%).
- LP pressure does not influence the post-LP headache.

## Supplemental Material

Supplementary figure -Supplemental material for Therapeutic lumbar puncture for headache in idiopathic intracranial hypertension: Minimal gain, is it worth the pain? Click here for additional data file. Supplemental material, Supplementary figure for Therapeutic lumbar puncture for headache in idiopathic intracranial hypertension: Minimal gain, is it worth the pain? by Andreas Yiangou, James Mitchell, Keira Annie Markey, William Scotton, Peter Nightingale, Hannah Botfield, Ryan Ottridge, Susan P Mollan and Alexandra J Sinclair in Cephalalgia

## Supplemental Material

Supplementary table -Supplemental material for Therapeutic lumbar puncture for headache in idiopathic intracranial hypertension: Minimal gain, is it worth the pain? Click here for additional data file. Supplemental material, Supplementary table for Therapeutic lumbar puncture for headache in idiopathic intracranial hypertension: Minimal gain, is it worth the pain? by Andreas Yiangou, James Mitchell, Keira Annie Markey, William Scotton, Peter Nightingale, Hannah Botfield, Ryan Ottridge, Susan P Mollan and Alexandra J Sinclair in Cephalalgia
