# Supplementary material for: Therapeutic lumbar puncture for headache in idiopathic intracranial hypertension: Minimal gain, is it worth the pain?
Source: Cephalalgia. 2018 Jun 17;39(2):245–53. doi: 10.1177/0333102418782192 (PMC6376596; doi:10.1177/0333102418782192)
Supplement: Supplementary figure -Supplemental material for Therapeutic lumbar puncture for headache in idiopathic intracranial hypertension: Minimal gain, is it worth the pain? [file Supplementary_figure.pdf]

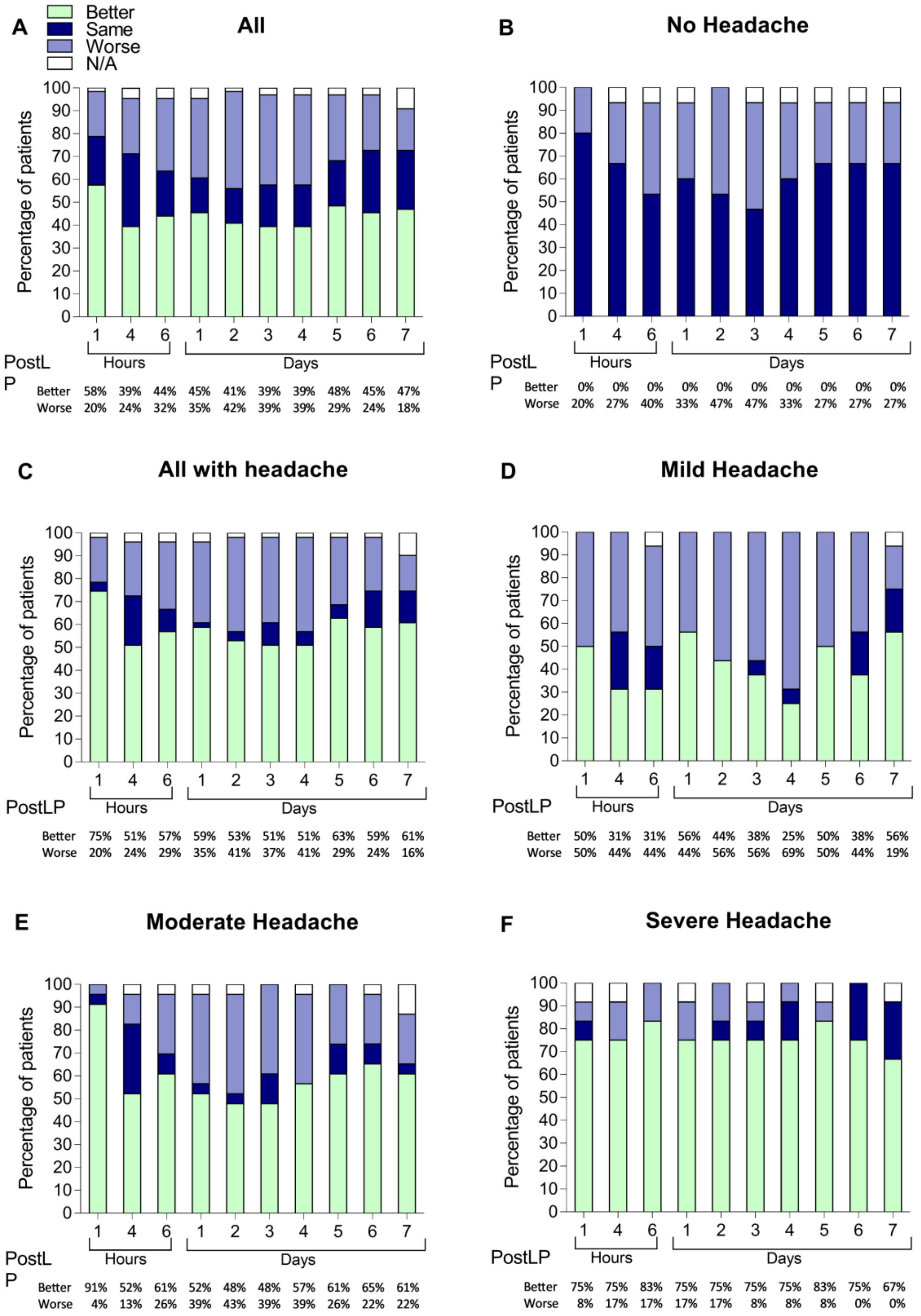

Supplementary Figure 1 Percentage of patients experiencing improvement or deterioration of headache classified according to pre-lumbar puncture (LP) headache severity. A) All patients, B) No headache (on the day of the LP, at the pre-LP time point), C) All with headache D) Mild headache pre-LP, E) Moderate headache pre-LP, F) Severe headache pre-LP.
