# Supplementary material for: Therapeutic lumbar puncture for headache in idiopathic intracranial hypertension: Minimal gain, is it worth the pain?
Source: Cephalalgia. 2018 Jun 17;39(2):245–53. doi: 10.1177/0333102418782192 (PMC6376596; doi:10.1177/0333102418782192)
Supplement: Supplementary table -Supplemental material for Therapeutic lumbar puncture for headache in idiopathic intracranial hypertension: Minimal gain, is it worth the pain? [file Supplementary_table.pdf]

Supplementary Table 1: Headache scoring responses at each time interval from total of 66 paper diaries.

Time Point	Hours				Days						
	0	1	4	6	1	2	3	4	5	6	7
**Responses**	66	66	63	63	63	63	64	64	65	65	66
